# The Story of the Insane from Year to Year

**Published:** 1897-07-24

**Authors:** 


### THE STORY OF THE INSANE FROM YEAR TO YEAR.

Tenth Series.

### SUNDERLAND BOROUGH ASYLUM.

Here the numbers rose during the year from 288 to 314. There were 116 first admissions, 6 readmissions, 43 recoveries, 7 discharged relieved, and 33 deaths. The recoveries stand at 43·8 per cent. of the admissions, and the deaths at 10·3 on the average number resident. Nineteen of the deaths were caused by cerebral or spinal diseases, 4 by consumption, and 2 by pneumonia. Seventeen of the year's admissions owed their insanity to various moral causes, 27 to intemperance in drink, and 44 to hereditary influence. This is only the second annual report of the asylum, and yet we learn that the male side is almost full, and that it would have been quite full had it not been for the discharge of harmless chronic patients to their own homes. Dr. Elkins very correctly points out that the list of such cases must necessarily be a limited one and that a great responsibility is incurred by discharging unrecovered patients, and, we may add, recovered ones also; but then the latter is imperative, while the former is optional. It is a curious fact, too, that the proximity of an asylum seems to increase the demand for asylum accommodation. Dr. Elkins tells us that there are twice as many patients sent to the Sunderland Asylum than were sent from the borough to the Durham Asylum. Our asylum system will never be complete until a block for private patients is erected in the grounds of every county and borough asylum. On this subject Dr. Elkins' remarks are very good. "There seems to me," he says "to be considerable hardship in this treatment of private patients capable of paying a moderate board. The rich can always find asylum accommodation and the poor can demand it." But for the middle classes, or rather the lower edge of the middle classes, there is hardly any place, and the inevitable consequence is that these patients drift into the wards of our public asylums, either as private patients or paupers. As already said, the asylum is almost new, and it is found to be insufficient in size, but we are also told that some parts of it are "neither suitable, adequate, nor convenient for the treatment of acutely sick patients." The asylum was visited by erysipelas, typhoid fever, septic pneumonia, and a considerable number of the patients and staff suffered from septic sore throat and anæmia, and these diseases were traceable to the defective state of the drainage. Several of the staff obtained the nursing certificate of the Medico-Psychological Association, and the committee have wisely decided to increase the wages of these. We venture to hope that Dr. Elkins and Dr. Yeates will carry their instructions far beyond what is required for this certificate. We are not a little pleased to notice that Attendant James Anderson was presented with a silver watch for an act of great bravery. The weekly cost per head was 8s. 10⅝d.—a low rate for so small an asylum.

